# Supplementary material for: Tailoring Heat Transfer and Bactericidal Response in Multifunctional Cotton Composites
Source: Nanomaterials (Basel). 2023 Jan 23;13(3):463. doi: 10.3390/nano13030463 (PMC9919448; doi:10.3390/nano13030463)
Supplement: Supplementary file 1 [file nanomaterials-13-00463-s001.zip › nanomaterials-2151412-supplementary.pdf]

nanomaterials

Supplementary Materials

Tailoring Heat Transfer and Bactericidal Response in Multifunctional Cotton Composites

Lilian Pérez Delgado [1,*], Adriana Paola Franco-Bacca [1], Fernando Cervantes-Alvarez [1], Elizabeth Ortiz-Vazquez [2], Jesús Manuel Ramon-Sierra [2], Victor Rejon [1], María Leopoldina Aguirre-Macedo [3], Juan José Alvarado-Gil [1] and Geonel Rodríguez-Gattorno [1,*]

[1] Merida Unit, Functional Materials Laboratory, Applied Physics Department, Center for Research and Advanced Studies (CINVESTAV), C.P. 97310 Merida, Mexico; adriana.franco@cinvestav.mx (F.-B.A.P.); fernando.cervantes@cinvestav.mx (C.-A.F.); vrejon@cinvestav.mx (R.V.); juan.alvarado@cinvestav.mx (A.-G.J.J.)
[2] Merida Unit, Laboratory of Applied and Molecular Microbiology, National Technological Institute of Mexico, C.P. 9711 Merida, Mexico; elizabeth.ov@merida.tecnm.mx (O.-V.E.); jesus.rs@merida.tecnm.mx (R.-S.J.)
[3] Merida Unit, Aquatic Pathology Laboratory, Marine Resources Department, Center for Research and Advanced Studies (CINVESTAV), C.P. 97310 Merida, Mexico; leopoldina.aguirre@cinvestav.mx
* Correspondence: lilian.perez@cinvestav.mx (D.L.P.); geonelr@cinvestav.mx (R.-G.G.)

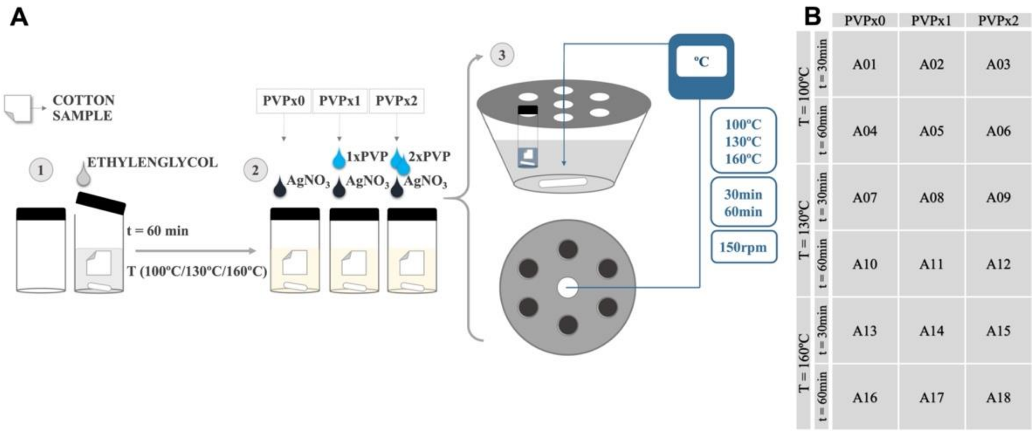

Figure S1. (**A**) Descriptive three steps diagram of the general methodology for the coating of AgNPs on textile fibers. The synthesis process was carried out in a self-design device with a glass base, metal cover, six holes for hexagonal distribution samples and a central one for the temperature sensor and vapor outlet. (**B**) Distribution of eighteen samples according to the selected reaction parameters.

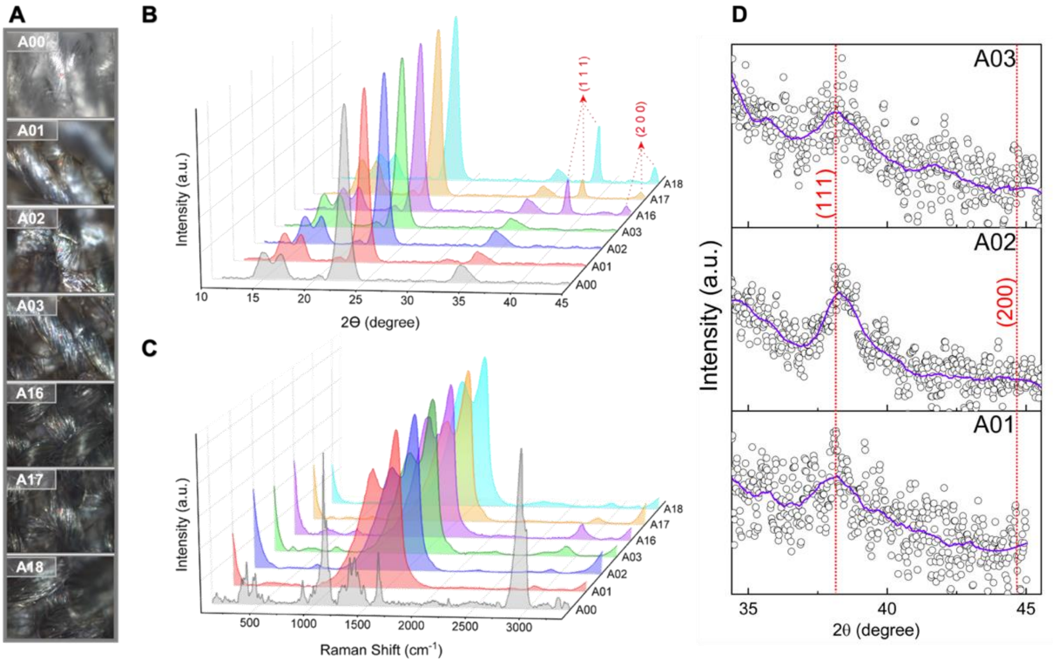

Figure S2. (**A**) Digital microscope images, (**B**) XRD and Raman (**C**) spectra of threads from bare cotton (A00) and six selected AgNPs@cellulose samples: A01 (T = 100°C, t = 30 min, PVPx0), A02 (T = 100°C, t = 30 min, PVPx1), A03 (T = 100°C, t = 30 min, PVPx2), A16 (T = 160°C, t = 60 min, PVPx0), A17 (T = 160°C, t = 60 min, PVPx1) and A18 (T = 160°C, t = 60 min, PVPx2). (D) magnification of the angular range where reflections of the metallic silver are expected, for samples A01, A02 and A03.

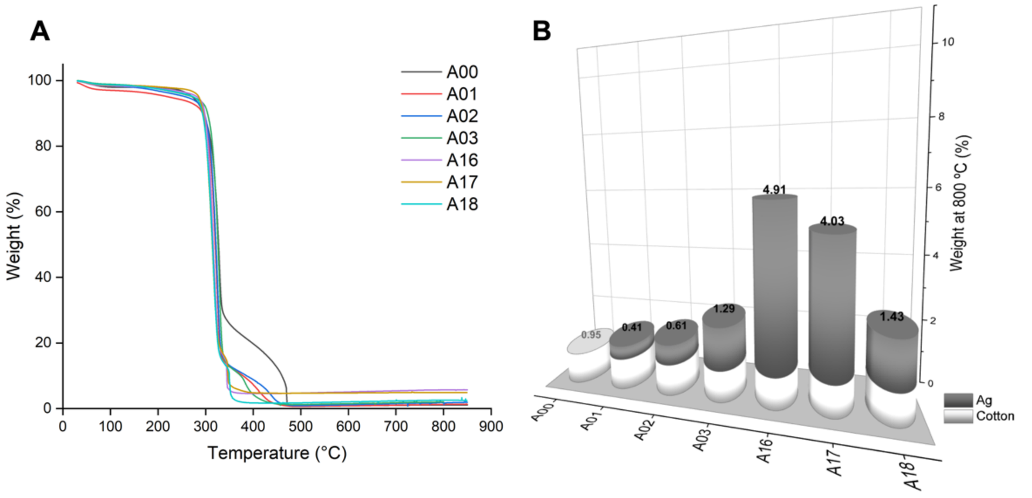

Figure S3. TGA curves (**A**) and percentage weight values at 800 °C. (**B**) of bare cotton (A00) and six selected AgNPs@cellulose samples: A01 (T = 100 °C, t = 30 min, PVPx0), A02 (T = 100°C, t = 30 min, PVPx1), A03 (T = 100°C, t = 30 min, PVPx2), A16 (T = 160°C, t = 60 min, PVPx0), A17 (T = 160 °C, t = 60 min, PVPx1) and A18 (T = 160 °C, t = 60 min, PVPx2).
